# Is there a ‘learning effect’ on serial lung function assessments?

**DOI:** 10.1113/EP093416

**Published:** 2025-12-14

**Authors:** Rie Skovly Thomsen, Iben Elmerdahl Rasmussen, Ronan M. G. Berg

**Affiliations:** ^1^ Centre for Physical Activity Research Copenhagen University Hospital – Rigshospitalet Copenhagen Denmark; ^2^ Department of Biomedical Sciences, Faculty of Health and Medical Sciences University of Copenhagen Copenhagen Denmark; ^3^ Department of Clinical Physiology and Nuclear Medicine Copenhagen University Hospital – Rigshospitalet Copenhagen Denmark; ^4^ Department of Clinical Medicine, Faculty of Health and Medical Sciences University of Copenhagen Copenhagen Denmark; ^5^ Neurovascular Research Laboratory, Faculty of Life Sciences and Education University of South Wales Pontypridd UK

Measurements of dynamic lung volumes and pulmonary diffusing capacity are used to assess lung function, both in the clinical setting, for example to diagnose, classify and monitor lung disease and treatment effects, and in mechanistic studies on lung physiology. However, anyone who has conducted lung function testing, whether as a lung function technician, a patient or an experimental subject, will know that the rather complex manoeuvres require considerable effort, motivation and a high degree of cooperation. An obvious question that is often raised when serial lung function assessments are reported is whether there is a ‘learning effect’, as also known from other related physiological measurements such as maximal oxygen uptake (Barron et al., 2014; Edgett et al., 2018) and one‐repetition maximum testing (Benton et al., 2009; Mendes Ritti‐Dias et al., 2011). That is, can an observed improvement in lung function merely reflect that participants have become better at performing lung function testing? The short answer is no! The longer answer is a little more gritty, as we shall see below, but the conclusion nevertheless remains the same.

Dynamic lung volumes, including forced expiratory volume in 1 s (FEV_1_) and forced vital capacity (FVC), are obtained by dynamic spirometry, while pulmonary diffusing capacity is typically measured by the single‐breath method. These parameters are among the most widely used lung function metrics, and all are crucial for clinical decision‐making. It is therefore a *sine qua non* that these metrics are comparable between different operators and lung function laboratories. For this reason, extensive efforts have been made by the European Respiratory Society (ERS) and the American Thoracic Society (ATS) to provide guidelines for equipment technical specifications and calibration procedures, as well as detailed recommendations on how tests should be performed (Graham et al., 2017, 2019). These include objective quality criteria based on the operator's assessment of how well the subject performs a given manoeuvre; that is, its ‘acceptability’ and ‘repeatability’.

In the recently updated ERS/ATS guidelines, the quality grading of a lung function test is expressed by assigning a letter grade (A, B, C, D, E, U or F for a reported value by dynamic spirometry; A, B, C, D or F assigned to each manoeuvre for pulmonary diffusing capacity), which informs how confident one can be in the given metric and thus how it should be weighed in clinical decision‐making (Graham et al., 2017, 2019). Although this focus on rigorous quality assessment was intended to ensure that tests are performed correctly to provide valid and reliable estimates, the question remains: could a clinically or physiologically meaningful ‘learning effect’ confound true change (or lack thereof) in lung function over time?

For dynamic spirometry, small familiarisation effects are well known, occurring mainly within a single visit, that is, between the first versus later blows. However, these intra‐session ‘learning effects’ are still exceedingly small when tests are performed according to a standard under supervision by experienced personnel. In 18,000 consecutive patients aged 20–90 years, 90% of the patients were able to reproduce both FEV_1_ and FVC within 5–6% within one session when testing was performed by experienced personnel (Enright et al., 2004). Intersession differences for repeated dynamic spirometry on the same day also appear to be of a similar magnitude (Kunzli et al., 1995). However, these data may to some extent be confounded by circadian effects (Goyal et al., 2019). In a more recent study, dynamic spirometry was repeated over several days in 30 healthy volunteers. Daily lung function testing was performed during the first week, followed by 3 weeks with weekly testing on the same weekday, and concluding with another week of daily testing (Krabbe et al., 2023). Curiously, higher FEV_1_ and FVC values were observed on day 3 and 4; however, this did represent any sort of learning effect, since it had ceased by week 3. In the same study, a financial incentive was introduced in a randomised fashion as a motivational strategy to achieve the highest dynamic lung volumes, but this had no effect.

As for the single‐breath technique for measuring pulmonary diffusing capacity, studies have noted substantial between‐day variability upon repeated testing, but not with any systematic differences in the reported metrics when quality control is applied (Drummond et al., 2008). Likewise, in our hands, when the single‐breath technique was used to measure pulmonary diffusing capacity (Hartmann et al., 2025; Madsen et al., 2023), no systematic increase was observed when measurements were repeated on separate days (two to ten days apart), as would have been expected if there was a learning effect. We found a smallest real difference between measurements of ∼1.0 mmol min^−1^ kPa^−1^ in both young and slightly older healthy individuals as well as in patients with COPD (Hartmann et al., 2025; Madsen et al., 2023).

While there are no systematic intersession differences in any of the metrics typically obtained by dynamic spirometry or the single‐breath technique, there appears to be a clear learning effect on the *quality* of lung function assessments. Large longitudinal studies involving repeated testing have shown that the within‐test variability between blows decreases over the first two to three visits, and that fewer manoeuvres are required to meet acceptability and repeatability criteria when performing dynamic spirometry (Bellia et al., 2000; Enright et al., 1991; Janssens et al., 2013). Similarly, for pulmonary diffusing capacity, intra‐session precision improves over consecutive manoeuvres as participants become more practised in the breath‐hold manoeuvre (Radtke et al., 2017). Accordingly, with repeated assessments, there is a gradual decrease in the number of manoeuvres needed to achieve acceptability and repeatability criteria over the first two to three sessions, after which the number of required manoeuvres per session reaches a plateau (Wise et al., 2007). It is important to note that lower test quality does not cause a systematic increase or decrease in any of the reported metrics; rather, it affects their accuracy and precision, thereby influencing variability at the group level and in longitudinal responses. This stresses the importance of not reporting only the lung function metrics, but also their quality, that is, by use of the ERS/ATS quality grading system.

So, to answer the question: is there a ‘learning effect’ on lung function measurements? There clearly appears to be one with regard to measurement quality. However, there is currently no firm evidence to suggest that this leads to any consistent intersession differences in the reported lung function metrics.

## AUTHOR CONTRIBUTIONS

Rie Skovly Thomsen: revisions, figure. Iben Elmerdahl Rasmussen: revisions, figure. Ronan M. G. Berg: Conception, first draft, revisions, supervision. All authors approved the final version of the manuscript and agree to be accountable for all aspects of the work in ensuring that questions related to the accuracy or integrity of any part of the work are appropriately investigated and resolved. All persons designated as authors qualify for authorship, and all those who qualify for authorship are listed.

## CONFLICT OF INTEREST

None of the authors have any competing interests to declare.

## FUNDING INFORMATION

The Centre for Physical Activity Research (CFAS) is supported by TrygFonden (grants ID 101390, ID 20045, ID 125132, and ID 177225). The funders had no role in study design, data collection and analysis, decision to publish, or preparation of the manuscript.
